# Climate Disaster and Cognitive Ability: Evidence From Wildfire

**DOI:** 10.3389/ijph.2024.1607128

**Published:** 2024-07-10

**Authors:** Ran Du, Ke Liu, Dangru Zhao, Qiyun Fang

**Affiliations:** ^1^ School of Economics, Huazhong University of Science and Technology, Wuhan, Hubei, China; ^2^ International Business School, Shaanxi Normal University, Xi’an, Shaanxi, China

**Keywords:** human capital, climate disaster, wildfire, cognitive ability, wind direction realization

## Abstract

**Objectives:**

We investigate the impact of wildfire disasters on cognitive health (i.e., thinking and language skills) in individuals aged 10 years and older using 2014 and 2018 wildfire and cognitive ability survey data from China.

**Methods:**

We distinguished wildfires in each county at different wind directions each day by exogenous wind direction changes, and analyzed the effects of wildfires on cognitive abilities through upwind and non-upwind wildfires.

**Results:**

Our analysis shows that for every 10-unit increase in upwind wildfires compared to non-upwind wildfires, respondents’ scores on word and math tests decrease by 0.235 and 0.236 standard deviations, respectively. Furthermore, we find that the impact of wildfire on cognitive ability is more pronounced in younger individuals, and those with lower defensive expenditures experience more severe impacts. Additionally, wildfires negatively affect individuals’ cognitive abilities by generating air pollution.

**Conclusion:**

Wildfires significantly reduce individuals’ cognitive abilities. Two recommendations are as follows: 1) governments should follow the principle of green development, introduce relevant regulations, and increase investment in adaptive technologies. 2) Individuals should raise awareness of climate hazards preparedness and strengthen defensive protection.

## Introduction

The rate and strength of wildfires are increasing due to climate change, leading to higher levels of carbon emissions, degraded air quality, and significant impacts on socioeconomics [1]. The Lancet Countdown to 2021 China Report shows a 24.5% increase in average annual wildfire risk in China from 2016 to 2020 compared to 2001–2005, with 20 provinces experiencing higher wildfire risk. Accurately portraying the economic and social costs of climate disasters is a major concern in academia. At the same time, cognitive ability has long been considered an important component of human capital and is crucial to decision-making, human behavior formation, and economic development [2, 3]. Clarifying the impact of climate disasters on human capital is of great significance for optimizing climate disaster governance policies and promoting stable economic growth in various countries.

In recent years, the issue of global warming has become more prominent, leading to an increase in the severity and consequences of climate disaster such as wildfires. Research in the field of economics has revealed significant harmful effects of wildfires on human health [4]. Wildfires can directly cause fatalities such as burns, heat exposure, and smoke inhalation, as well as a significant economic burden through the release of fine particulate matter and other pollutants leading to increased rates of respiratory and cardiovascular disease, and psychological disorders such as anxiety and depression, which can increase long-term medical costs [5, 6]. Additionally, some scholars have further estimated the economic losses associated with wildfire disasters on human health. According to the research by Johnston et al. (2021), the health costs caused by the 2019–2020 Australian wildfires amounted to AUD 1.95 billion [7]. In addition to analyzing the impact on human health, economists have increasingly focused on various economic consequences of wildfire disasters. These include individual defensive behavior and willingness to pay [8], the outdoor recreation economy [9], fluctuations in housing prices [10, 11], the value of local facilities and services [12], and mortgage loan risk [13]. Recently, some researchers have started to examine the impact of wildfires on human capital. However, existing research mainly focuses on exploring the impact of wildfires on labor supply and productivity. For instance, Borgschulte et al. discovered that every extra day of wildfire exposure results in approximately a 0.1% reduction in quarterly labor income [14].

In addition to impacting economic activity and physical health, wildfires can also have a direct impact on human cognitive abilities, resulting in increased health costs and loss of human capital [14, 15]. While there is limited focus on the relationship between wildfire disasters and cognitive health, research is increasing on the impact of straw burning, a form of fire pollution from wildfires, on cognitive health. Zivin et al. discovered that as the difference in fires between upwind and downwind increased by one standard deviation, the total test result decreased by 1.42% [16]. Lai et al. found that PM2.5 emissions from upwind straw burning contributed to decreased cognitive health [17]. Based on the aforementioned empirical research, it is theoretically possible that wildfire can also have a significant impact on human cognitive abilities. In particular, recent studies have found that wildfires increase air pollution levels [14, 18]. Furthermore, previous studies have shown that air pollution can lead to damage to the nervous system and impair brain function as a result of the potential neurotoxicity of air pollutants [17, 19–21]. Air pollutants such as particulate matter and nitrogen dioxide have been shown to trigger neuroinflammation and oxidative stress in the brain, leading to chronic inflammation, cell damage, and ultimately cognitive decline [22]. The direct neurotoxic effects of certain pollutants, along with vascular damage and disruption of neurotransmitter systems, further exacerbate these effects. Consequently, this can lead to diminishing individual cognitive abilities and productivity [23–25]. Therefore, we argue that wildfire disasters may impact individual cognitive health through air pollution.

**FIGURE 1:**
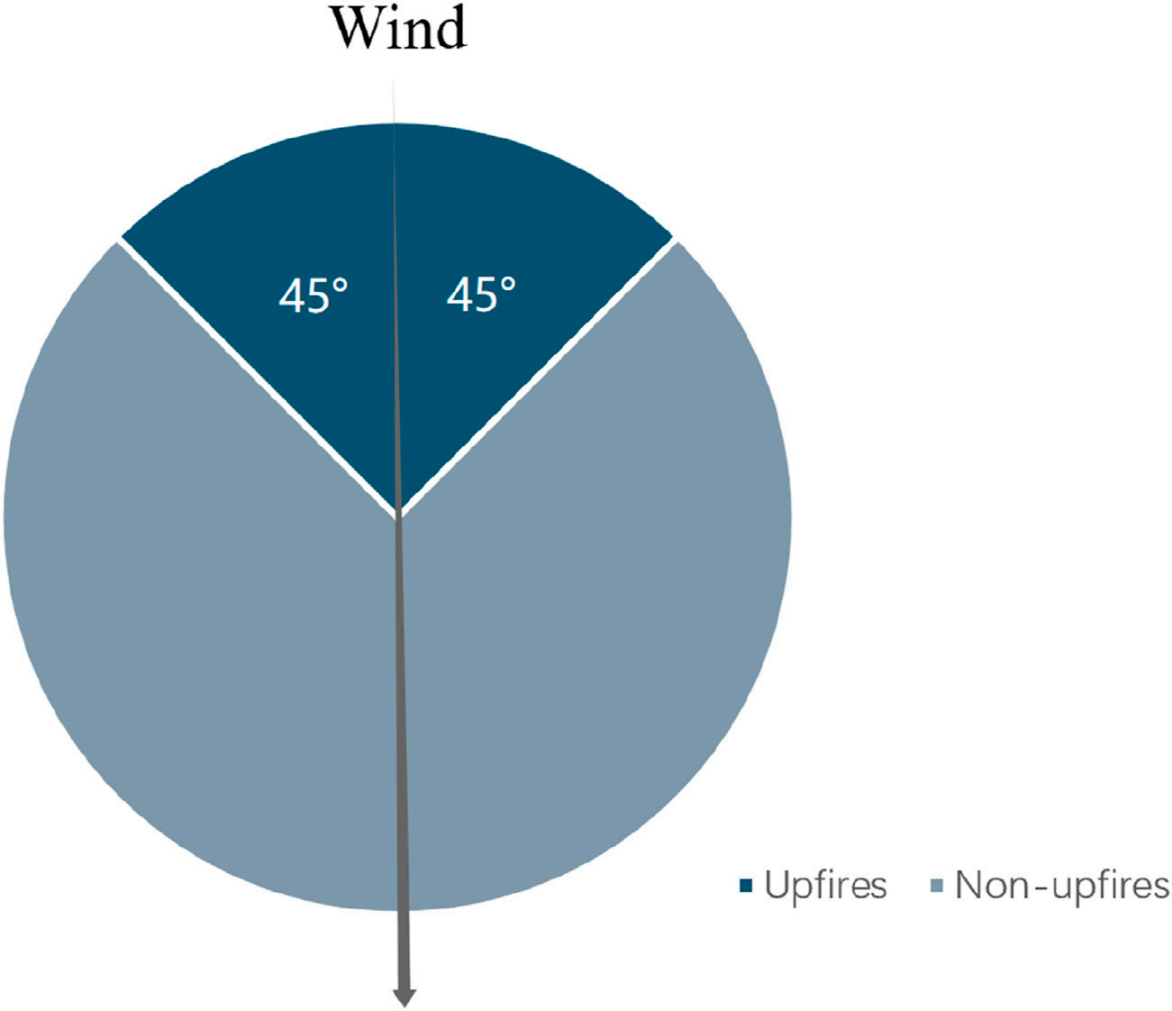
Wildfires under different wind direction types (China. 2023).

However, the following challenges exist in current research on the effects of wildfire on cognitive health. First, previous studies have focused on some of the areas where large wildfires have occurred, and less on the impacts of less intense wildfires that are farther away from cities [4]. To address data limitations and data accuracy issues, we used VIIRS satellite wildfire monitoring data [26, 27]. Compared to previous wildfire survey data, this data provides a more comprehensive representation of nighttime and small-scale wildfires, and therefore allows for a more accurate monitoring of scattered or short-burning wildfire events [28]. Second, it is difficult to study the pollution effects of wildfires on health purely by excluding other economic factors that affect cognitive ability through causal identification methods. Individual health can be affected by a variety of socio-economic factors, individual behavioral choices, or other environmental pollution factors that can also influence the pollution effects of wildfires [29]. Failure to exclude other influences can lead to biased estimates. To address this issue, this study adopts the approach of Rangel and Vogl [29], which utilizes exogenous changes in different wind directions for empirical analysis. We are specifically comparing the difference in cognitive ability impacts between areas with prevailing winds and those without. Taking the county administrative center point as the center of the circle, with a radius of 50 km, the daily maximum downwind wind direction is connected to the center of the circle, and the area with a 45° between its right and left side is the upwind direction, see the shaded area in Figure 1, while the other areas are the non-upwind direction areas, see the blank area in Figure 1. This discrepancy is interpreted as the causal effect of wildfires on cognitive ability, taking into consideration confounding factors. The approach assumes that people in areas with prevailing wind directions experience varying wildfire pollution levels but are equally affected by other confounding factors, such as being in counties with the same economic conditions or other pollutants. At the same time, the daily wind direction and the location of fire point sources are frequently changing across districts and counties, which helps to further distinguish pollution effects from confounding effects and reduce the problem of selection bias that has arisen from the selection of stationary sources (e.g., factories) in the previous literature.

Therefore, we gathered daily wildfire data for each county in China in 2014 and 2018. To analyze the impact of wildfires on cognitive ability, we employed different wind direction models.

This paper has the following potential research contributions. Firstly, we expand on the literature about the impact of climate change on cognitive health and human capital. By focusing specifically on the impact of wildfire disasters, this study sheds light on the external costs associated with such events and offers valuable insights into their wide-ranging consequences. While many natural disasters typically result in localized damage, the empirical findings presented in this paper highlight the potential for significant harm even in areas far removed from the fire’s location. This generates substantial negative externalities and provides a novel perspective for comprehending the economic and social costs of climate-related disasters. Secondly, we add to the research literature on the determinants of cognitive ability. Existing literature has primarily focused on micro-level factors such as household and individual characteristics [30–32] while giving less attention to the influence of macro-environmental factors on cognitive ability. In particular, the existing studies on how external environmental factors impact cognitive ability has mainly focused on policy reform and environmental pollution [21, 33, 34]. We explore an important gap in existing literature by offering valuable insights into this underexplored area. Thirdly, this paper employs a more detailed approach to measuring individuals’ cognitive aptitude. By assessing cognitive ability through verbal and mathematical tests, this study enables a more comprehensive empirical analysis.

## Methods

### Variable Selection

This paper selects wildfire data and the China Family Panel Studies Database from counties in China in 2014 and 2018. The reasons for selecting these years are as follows: 1) The VIIRS monitor began releasing complete annual fire point data in 2013, making 2014 an appropriate starting year for this study. 2) In November 2018, the Chinese government formed a new national comprehensive firefighting and rescue team, effectively alleviating fire hazards. We chose 2018 as the cut-off year for the study in order to minimize the potential impact of the policy intervention on the results. Samples with missing or negative values for all variables are excluded from the analysis.

#### Cognitive Ability

This survey data employs two types of tests to assess the cognitive ability of respondents: a word group test and a math test. The cognitive ability calculator is based on the 2010 cognitive test design method. Such a selection ensures that the results are comparable. See Supplementary Appendix 1 for specific test methods. This paper standardizes all cognitive ability test indicators. Supplementary Table S1 describes the characteristics of the respondents.

#### Wildfire

Wildfire refers to the burning of biomass in wild ecosystems, such as forest wildfires and grassland wildfires. It can have a significant impact on natural ecosystems and global climate change [18, 35, 36]. We draw on the studies of Hantson [26], Marcos [27], and Csiszar [37], and we obtained high-resolution satellite wildfire monitoring data from the Visible Infrared Imaging Radiometer (VIIRS) fire point product dataset [38]. Firstly, We obtained latitude and longitude data from VIIRS for daily wildfire locations in the China region and used ArcGIS software to obtain the specific address of each fire point using an inverse coding method. Secondly, we follow the research of Rangel and Vogl [29], we summed the number of wildfires per day within 50 km of each county’s administrative center by year to form county-year data. Finally, the number of wildfires was divided by 10 for ease of interpretation of the results.

We also need to use detailed wind direction data to measure wildfires in different wind directions. We measure the daily wind direction in each county using the wind direction at the weather station’s maximum wind speed. We excluded counties with two or more weather stations based on data availability and applicability. The wind direction data is sourced from the daily surface climate dataset of the China Meteorological Data Center.

#### Control Variables

Meteorological control variable data were also obtained from the daily surface climate dataset of the China Meteorological Data Center. It mainly includes average precipitation (unit: mm), average temperature (unit: °C), average wind speed (unit: m/s), and average humidity (unit: %) in each county. The raw raster data is interpolated to grid data using inverse distance weighting (IDW) interpolation [39]. Regarding the selection of other individual control variables in the baseline regression, this paper follows the practice of Lai et al. [17]. Indicators such as gender, total family income, social status, and medical insurance are selected as individual-level control variables.

#### Mechanism Variables

This paper selects air pollution as a mechanism variable. This study uses AQI, PM2.5, PM10, and NO_2_ to measure air pollution. The original data comes from the National Urban Air Quality Dissemination Site.

The definition of the main variables is shown in Supplementary Table S2. The descriptive statistics of the main variables are shown in Supplementary Table S3.

### Model

We separate the wildfires in each county by wind direction on a daily basis. The model is set as follows:

$$Y_{it}=\beta_{0}+\beta_{1}{Upfire}_{it}+\beta_{2}{Nonupfire}_{it}+\beta_{3}{Control}_{it}+\pi_{t}+\tau_{ph}+\varepsilon_{it}$$ (1)

Where i represents the individual, t represents time, h represents the household, and p represents the province. Y represents the cognitive ability, Upfire and Nonupfire represent the number of upwind and non-upwind wildfire, Control represents other control variables that include precipitation temperature, wind speed, humidity, gender, total family income, social status, and medical insurance. $\beta_{0}$ denotes the constant term, $\beta_{1}$ indicates the magnitude of the effect of upwind wildfires on cognitive ability, $\beta_{2}$ indicates the magnitude of the effect of non-upwind wildfires on cognitive ability, $\beta_{3}$ denotes the set of coefficients for the effect of each control variable on cognitive ability. $\pi_{t}$ is the time-fixed effect, $\tau_{ph}$ is the Upfires-Nonupfires fixed effect of province and household, and $\varepsilon_{it}$ is the random error term.

## Results

### Effects of Wildfire Disaster on Cognitive Abilities

Table 1 presents the empirical results based on Eq. 1. Columns (1) and (2) display the estimated results for the word test scores. The results in column (1) indicate a significant reduction in individual word test scores with an increase in wildfire incidents when only meteorological control variables are considered. Specifically, relative to non-upwind wildfires, an increase of 10 upwind wildfires reduces individual word test scores by 0.216 standard deviations. In column (2), after further controlling for individual characteristics, relative to non-upwind wildfires, an increase of 10 upwind wildfires reduces word test scores by 0.235 standard deviations. Columns (3) and (4) in Table 3 show the impact of wildfire on individual math test scores. Column (3) shows that, without controlling for individual characteristics, a 10th increase in the difference between upwind and non-upwind wildfires is associated with a decrease of 0.217 standard deviations in math test scores. Column (4) shows that after controlling for individual characteristics, the cumulative effect estimate coefficient is −0.236. Our empirical findings suggest that wildfires can impair individuals’ cognitive abilities, highlighting the importance of implementing measures to prevent and mitigate the impacts of climate-related disasters on cognitive function.

**TABLE 1:** Effects of wildfire disaster on cognitive abilities (China. 2023).

	(1) Wordtest	(2) Wordtest	(3) Mathtest	(4) Mathtest
Upfires	−0.099***	−0.111***	−0.087*	−0.099**
	(−3.109)	(−3.519)	(−1.895)	(−2.139)
Nonupfires	0.118***	0.124***	0.130**	0.137**
	(3.218)	(3.420)	(2.418)	(2.520)
Upfires-Nonupfires	−0.216***	−0.235***	−0.217**	−0.236***
	(−3.284)	(−3.582)	(−2.217)	(−2.383)
Observations	6,700	6,700	6,700	6,700
*R* ^2^	0.476	0.526	0.444	0.490
Meteorological control variables	Y	Y	Y	Y
Individual control variables	N	Y	N	Y
Household FE	Y	Y	Y	Y
Province-Year FE	Y	Y	Y	Y

Note: The numbers in parentheses are t-values clustered at the household level. *, **, and *** indicate significance at the 10%, 5%, and 1% levels, respectively. The same applies to the table below.

### Robustness Checks

To enhance the validity and credibility of the empirical results, we conduct robustness tests from the perspectives of different measurements of dependent and independent variables, sample selection, and different model specifications, respectively. We show here only the test results of the instrumental variables approach; other robustness tests are in the Supplementary Material.

#### Instrumental Variables Method

This study addresses potential endogeneity concerns by utilizing ventilation as an instrumental variable for analyzing the impact of wildfires. In the standard air pollution box model, the ventilation coefficient is calculated as the product of the wind speed and the mixing height of the boundary layer, determining the rate of pollution dispersion [40]. We utilize wind speed and boundary layer height data from the ERA-Interim database of the European Centre for Medium-Range Weather Forecasts. The speed of the wind affects how pollutants spread horizontally, while the height of the boundary layer determines how they spread vertically. Higher ventilation coefficients not only have a direct impact on wildfire fire intensity but also transport contaminant particles from wildfire smoke to farther and higher regions with more severe impacts [41, 42]. Therefore, there is a correlation between ventilation coefficients and wildfires. Large-scale weather systems impact ventilation coefficients, which can be regarded as exogenous to individuals’ health and qualify as instrumental variables.

Wildfires are measured by dividing the total number of wildfires within a 50-kilometer radius of the administrative center by 10. Columns (1) to (3) of Table 2 report the full-sample empirical results using instrumental variables method. The results show that the cross-multiplication terms of the wildfire and instrumental variables were significantly positive and the Kleibergen-Paap Wald F statistic values were 70.785 in the first stage, proving there was no weak instrumental variable problem. The results of the second phase show that word and math test scores decreased by 0.149 and 0.248 standard deviations when wildfires increased by every 10th occurrence. This suggests that after reducing endogenous problems, an individual’s cognitive abilities can still be affected by wildfire.

**TABLE 2:** Instrumental variables method (China. 2023).

	(1) Ventilation	(2) Wordtest	(3) Mathtest
Wildfire	0.026***	−0.149*	−0.248***
	(6.779)	(−1.867)	(−2.619)
Observations	6,700	6,700	6,700
Kleibergen-Paap rk Wald F statistic	70.785		
Meteorological control variables	Y	Y	Y
Individual control variables	Y	Y	Y
Household FE	Y	Y	Y
Province-Year FE	Y	Y	Y

### Heterogeneity Analysis

First, there are significant differences between different age groups in their own physical fitness, escape skills, and level of knowledge about wildfires, which directly affects an individual’s ability to cope when faced with a fire [43, 44]. In addition, there are significant differences in attention and memory capacity, executive functioning, and abstract thinking across ages, which can affect an individual’s ability to perceive and cope with the environment. Thus, the effect of wildfires on an individual’s cognitive ability can be influenced by individual age heterogeneity. Second, defensive spending can reduce the duration and extent of an individual’s exposure to contaminants, thereby reducing the risk of mental illness. At the same time, defensive behaviors can help individuals improve their ability to cope with disasters, which can help individuals reduce their sense of maladjustment and improve their quality of life in environments that are more contaminated by wildfire, thus protecting their cognitive health. Thus, defensive spending is an effective way to constrain the health costs of wildfire pollution. Therefore, we also explored the heterogeneous effects in terms of individual defensive expenditures.

This article analyzes two aspects of heterogeneity: the age of the respondents and their defensive spending. Table 3 demonstrates the results of the heterogeneity test. All respondents were divided into middle-aged and elderly people over 50 years old and other non-middle-aged and elderly respondents according to age differences. The study found that wildfires had a greater negative impact on the cognitive abilities of young people, with each 10th upwind wildfire resulting in a 0.325 and 0.461 standard deviation reduction in young people’s scores on word and math tests, respectively. This finding is consistent with He [45]. The possible reasons are that, firstly, individuals under 50 years old spend more time outdoors, thus increasing the risk of direct and indirect harm from wildfire, secondly, older people have longer life spans and richer experiences, making them better able to adapt to the impact of natural disasters, finally, in the rapidly developing information age, young people can obtain disaster information more timely, thus easily generating stronger negative emotional pressure [46].

**TABLE 3:** Heterogeneous effects of wildfires on cognitive abilities (China. 2023).

Panel A	(1) Wordtest	(2) Mathtest	(3) Wordtest	(4) Mathtest
	**Age ≤ 50 years old**		**Age > 50 years old**	
Upfires	−0.174***	−0.227***	0.053	0.200
	(−4.032)	(−3.044)	(0.825)	(1.365)
Nonupfires	0.151***	0.234**	−0.013	−0.187
	(3.044)	(2.466)	(−0.153)	(−1.051)
Upfires-Nonupfires	−0.325***	−0.461***	0.0657	0.387
	(−3.611)	(−2.750)	(0.455)	(1.201)
Observations	3,844	3,844	2,856	2,856
*R* ^2^	0.581	0.538	0.740	0.893

Panl B	Low defensive expenditure		High defensive expenditure	
Upfires	−0.332***	−0.454***	−0.016	−0.054
	(−3.347)	(−4.074)	(−0.215)	(−0.991)
Nonupfires	0.380***	0.554***	0.091	0.107*
	(3.334)	(4.202)	(0.945)	(1.946)
Upfires-Nonupfires	−0.712***	−1.008***	−0.107	−0.161
	(−3.368)	(−4.178)	(−0.719)	(−1.555)
Observations	2,930	2,930	3,770	3,770
*R* ^2^	0.580	0.542	0.956	0.496
Meteorological control variables	Y	Y	Y	Y
Individual control variables	Y	Y	Y	Y
Household FE	Y	Y	Y	Y
Province-Year FE	Y	Y	Y	Y

The database’s investigators conduct healthcare expenditures surveys by asking respondents to answer the question, “In the past 12 months, how much did your household spend on expenditures that included fitness and exercise and the purchase of related product equipment and healthcare products?”. We divide respondents into different defensive expenditure groups according to their health expenditures. If a respondent’s health expenditure is greater than or equal to the average level of health expenditure, the individual is classified as a s high defensive expenditure group, and *vice versa* for the low defensive spending group. The results are shown in Panel B in Table 3. Relative to non-upwind wildfires, every 10th increase in upwind wildfires will lead to a significant decrease of 0.712 and 1.008 standard deviations in word and math test scores, respectively, for the low-defense spending group. This effect is greater for individuals with low defensive expenditures than for individuals with high defensive expenditures. The government and society should pay attention to and emphasize the group with low defensive expenditures, and guide and encourage this group to take active disaster prevention measures.

### Mechanism Analysis

We suggest that wildfire hazards may affect individuals’ cognitive abilities through the influence channel of air pollution. This is because smoke emitted by wildfires contains a substantial quantity of hazardous particles, including PM2.5 and nitrogen oxides. Primary pollutants can react in the atmosphere to form secondary pollutants like ozone, affecting regional air quality and public health [18]. Furthermore, previous studies have highlighted the potential neurotoxicity of air pollutants, which can harm the central and peripheral nervous systems, contribute to respiratory and cardiovascular ailments, and lead to a decline in cognitive ability. Therefore, we suggest that wildfire hazards can in turn affect the cognitive abilities of individuals by producing air pollution.

This article still uses a setting similar to Eq. 1 and replaces the explained variable with an air pollution index. The results are shown in Table 4. Specifically, compared with non-upwind wildfires, for every 10 additional upwind wildfires, PM2.5, PM10, AQI, and NO_2_ increased by 19.5%, 21.1%, 14.7%, and 22.2%, respectively.

**TABLE 4:** Mechanism test of the impact of wildfire disaster on cognitive abilities (China. 2023).

	(1)	(2)	(3)	(4)	(5)	(6)
	**Air pollution**					
	**PM2.5**	**PM10**	**AQI**	**NO_2_**	**Black_carbon**	**Organic_carbon**
Upfires	0.097***	0.108***	0.073***	0.134***	0.051***	0.044***
	(13.450)	(24.103)	(20.303)	(4.663)	(13.474)	(16.982)
Nonupfires	−0.098***	−0.103***	−0.074***	−0.087**	−0.059***	−0.050***
	(−10.358)	(−17.098)	(−15.438)	(−2.234)	(−10.881)	(−13.889)
Upfires-Nonupfires	0.195***	0.211***	0.147***	0.222***	0.110***	0.094***
	(11.75)	(20.37)	(17.72)	93.269)	(11.98)	(15.24)
Observations	6,700	6,700	6,700	6,700	6,700	6,700
*R* ^2^	0.997	0.998	0.998	0.992	0.999	0.999
Meteorological control variables	Y	Y	Y	Y	Y	Y
Individual control variables	Y	Y	Y	Y	Y	Y
Household FE	Y	Y	Y	Y	Y	Y
Province-Year FE	Y	Y	Y	Y	Y	Y

Notes: PM2.5 refers to particulate matter in the atmosphere with a diameter less than or equal to 2.5 μm. PM10 is particulate matter with a diameter equal to less than 10 μm. The Air Quality Index (AQI) is a non-linear dimensionless index that quantitatively describes air quality conditions. The larger the value, the more serious the air pollution condition and the greater the health hazard to human beings. Nitrogen dioxide (NO_2_) is one of the oxides of nitrogen, a reddish-brown gas with an irritating odor at room temperature.

In addition, Akagi found that incomplete combustion of biomass fires produces a large amount of black carbon and organic carbon, which are the main components of air pollution particles, such as PM2.5, and aggravate air pollution levels [47]. This article further studies the impact of wildfire disasters on black carbon and organic carbon, for which raw data are obtained from the M2T1NXAER dataset. The results are shown in columns (5) and (6) of Table 4. Black carbon and organic carbon increased by 11.0% and 9.4%, respectively, for every 10th increase in upwind wildfires compared to non-upwind wildfires. The results all suggest that wildfires can affect human cognitive abilities by producing air pollution. The finding underscores the public health risks associated with wildfires and air pollution. Understanding the effect of wildfires on cognitive abilities highlights the need for measures to protect public health during and after wildfire events.

## Discussion

### Conclusion

In the context of global climate change, it is essential to effectively address the adverse economic and social impacts of climate disasters. We investigate the effects of wildfire disasters on cognitive abilities using satellite-monitored wildfire data and cognitive ability survey data. The results show that exposure to wildfires leads to a significant reduction in the cognitive abilities of individuals. This finding underscores the serious public health implications of wildfires. The observed negative outcomes align with previous research on the adverse health effects of climate change and disasters [17, 21, 48–50]. Our research has also shown that wildfires produce large amounts of air pollution particles that can affect an individual’s cognitive abilities. Wildfires release a complex mixture of air pollutants, including particulate matter, volatile organic compounds, and solid particulate matter. The harmful pollutants can deeply penetrate the respiratory system, entering the bloodstream and potentially reaching the brain, which can have an impact on cognitive function [51]. Particulate matter, especially ultrafine particles (PM2.5), can have significant neurotoxic effects, impacting respiratory and cardiovascular health, as well as cognitive function. The cognitive impacts of wildfires have far-reaching consequences, affecting not only immediate wellbeing but also long-term health outcomes and community resilience in the face of natural disasters. Understanding these impacts is crucial for developing effective public health policies, and disaster response strategies, and enhancing community resilience in light of the disasters caused by climate change.

The results of our heterogeneity analysis indicate that younger individuals are more susceptible to the negative effects of wildfires on cognitive health. This could be attributed to factors such as still-developing physiological systems, longer life expectancy post-exposure, and increased time spent outdoors. Understanding age-specific vulnerabilities is crucial for designing effective public health interventions and policies aimed at protecting vulnerable populations from the health impacts of climate hazards. Moreover, our study underscores the role of socioeconomic factors in determining vulnerability to the health impacts of wildfires. Individuals with lower defensive expenditures, likely reflecting lower socioeconomic status, experienced more severe impacts on their cognitive and other health. Socioeconomic factors such as income and access to healthcare and resources play a critical role in determining an individual’s ability to mitigate and recover from the health impacts of climate hazards [52, 53]. Therefore, policies aimed at reducing vulnerability to the health impacts of wildfires should address underlying socioeconomic inequalities.

### Implications

Our study has dual policy implications. First, the findings highlight the importance and urgency of climate disaster governance. Given the substantial negative externalities that wildfire disasters can impose on individuals and society as a whole, governments need to enhance their early warning and response capabilities in the face of climate disasters and adopt proactive measures to mitigate their impacts. These measures may include but are not limited to, strengthening regulations pertaining to climate disasters and increasing investment in the research and development of adaptive technologies. Additionally, both individuals and the government should enhance their awareness of precautionary measures and defensive expenditure protection to minimize damage caused by wildfires. Second, developing economies like China are at a critical stage of transitioning to high-quality development. Cognitive ability, as a crucial component of human capital, plays a key role in enhancing individual and societal productivity and innovation capacity, thereby driving technological advancement and economic growth. However, climate disasters have had a significant impact on human capital, hindering economic transformation and upgrading. It is therefore imperative to fully embrace the principles of green development, effectively respond to climate disasters, actively pursue low-carbon development pathways, and mitigate the detrimental effects of climate change. By doing so, we can unleash the potential and value of human capital and promote long-term stable economic development.

Compared to previous studies, our study used satellite remote sensing to monitor wildfire data, including small-scale, nighttime, and away-from-urban-areas wildfires, allowing for a more accurate measure of episodic or short-duration wildfire events and more comprehensive and detailed data. The research shortcoming is that we used VIIRS 375-meter fire monitoring data, which may be affected by inversion accuracy, transit time points, and external factors such as lightning and cloudiness to include fire points from planned fires such as agricultural waste. Therefore, future research needs to further employ higher resolution remote sensing imagery with higher accuracy and more detailed land use classification data to extract wildfire ignition point data to improve the accuracy of the data samples. In addition, the cognitive ability survey data we used was limited to 2 years of data, 2014 and 2018, and future research could consider using survey data from a longer period.

## Data Availability

All authors ensure that all data and materials as well as software application or custom code support their published claims and comply with field standards.
