# Supplementary material for: Climate Disaster and Cognitive Ability: Evidence From Wildfire
Source: Int J Public Health. 2024 Jul 10;69:1607128. doi: 10.3389/ijph.2024.1607128 (PMC11266011; doi:10.3389/ijph.2024.1607128)
Supplement: Supplementary file 1 [file DataSheet1.docx]

**Variable Selection**

**Cognitive ability**

Table S1 shows the differential performance of respondents with different characteristics on the word test and the math test. The detailed descriptive statistical analysis allows us to observe the distribution and trend of the explanatory variables across the different characteristics. The results of the test for differences in means between the different characteristic groups indicate that the observed differences in cognitive test scores between gender, age, marital status, educational level, health status, social status, employment status, and region are statistically significant and are not due to random variation.

The first set of characteristics is the gender of the respondents, of which 50.85% are female and 49.15% are male. The wordtest score of female respondents was 14.07, which is lower than the mean wordtest of 18.54 for males, indicating that males on average perform better than females on the wordtest. Similarly, the mean mathtest score for females was 7.613, which was lower than the male mean of 10.09.In addition, the standard deviation of both wordtest and mathtest scores for males was smaller than that for females, indicating that the distribution of scores for males was more concentrated than that for females in both cases.

In the second group we described the grouping by age of the respondents according to the criteria for the division in the heterogeneity analysis section. The mean score on the word test for the 50 and under group was 20.35 with a standard deviation of 9.863, relative to the 50 and over group, indicating higher and more concentrated scores for this group. The mean score on the math test was 11.10 with a standard deviation of 6.191, which is more evenly distributed. Although the mean score of 5.769 with a standard deviation of 5.542 on the math test for the 50+ age group was also relatively evenly distributed, performance was generally lower.

In terms of the marital status of the respondents, the vast majority of the respondents were married (79.54%), and the mean and standard deviation of the cognitive test scores of the married group were lower than those of the unmarried group. This difference may be attributed to unmarried individuals having more time and energy to invest in learning and self-improvement, while married individuals may be more involved in family responsibilities.

The educational level of the respondents is shown in Table S1 and Figure S1, with 1,175 people having received a high school diploma and above, accounting for 17.54% of the number of respondents. Overall, the higher the level of education, the higher the cognitive test score scores, especially for the group that had obtained a college degree and above. For example, the average wordtest score for those with a college education was 27.93, with a median of 29. the average mathtest score was 17.22, with a median of 18, which is much higher than the average and median for the illiterate and semi-illiterate groups.

In Table S1 and Figure S2, it is evident that 851 individuals, accounting for 12.7% of the population, consider themselves to be very healthy. This segment represents a relatively small portion of the population. The number of people in the “More healthy” state is 2,444, accounting for 36.48%, and most of them feel that they are in a sub-healthy state, i.e., they do not have any major illnesses, but there are some symptoms such as discomfort and anxiety. In addition, the cognitive test scores of those in better health were higher. For example, those who self-assessed as “very healthy” had a mean score of 18.48 in words and 10.31 in math, much higher than the unhealthy group's scores of 9.861 and 5.271.This suggests that good physical health helps to maintain and improve cognitive ability, while poor health may lead to cognitive decline.

The social status of an individual can be expressed on a scale of 1 to 5, where 1 means very low status and 5 means very high status. Table S1 and Figure S3 show that out of 2944 respondents, majority of them (43.94%) have medium social status. These respondents scored the highest mean scores of 17.50 and 9.635 on the word test and math test respectively. The standard deviation of these two scores was 11.07 and 6.541 respectively, indicating a moderate level of dispersion. This is because higher socio-economic status tends to be associated with higher levels of education, employment and better health. The combination of these factors may influence cognitive ability test scores, thus highlighting the important impact of socioeconomic status on cognitive ability.

According to Table S1 and Figure S4, 43 are in unemployed status, 4889 are in in employment status, and 1365 are withdrawal from the labor market status. The “in employment” group, which comprised 73.7% of the population, had a mean score of 16.08 on the wordtest, with a median of 19 and a standard deviation of 10.78, indicating moderate and evenly distributed performance. In contrast, individuals who exited the labor market had a mean score of 13.00 on the word test, a median score of 12, and a standard deviation of 11.21, indicating lower and more dispersed performance. In the math test, the mean score for the employed group was 8.640 with a median of 8 and a standard deviation of 6.155, which also shows a more even distribution of scores. On the other hand, the group exiting the labor market had a mean score of 6.855 and a median of 6 on the mathtest. Compared to those who exited the labor market, the employed performed much better on both the word and math tests. In terms of the employment status of the respondents, it do not show a strong correlation with cognitive test scores. However, both wordtest and mathtest scores were lower for the withdrawal from the labor market group, possibly due to the fact that the majority of this group are likely to be older adults who have dropped out of the labor market.

From Table S1 and Figure S5, it can be seen that there were 3979 respondents or 59.39% of the total in the western region and 2380 respondents or 35.52% of the total in the eastern region. In terms of cognitive tests, respondents from the Western region scored an average of 15.62 and 8.489 points on the word and math tests, respectively. In contrast, respondents in the Eastern region scored an average of 17.02 and 9.198 points on the vocabulary and math tests, respectively. This difference may be attributed to unequal distribution of educational resources, varying levels of economic development, and cultural and social influences. The lower cognitive ability in the western region may be due to the relative lack of educational resources, slow economic development, and lower household income. On the contrary, the higher level of economic development in the eastern region, the abundance of educational resources, and the higher importance attached to education by families and society may explain the higher cognitive ability of their respondents. These findings suggest that there is a significant gap in educational achievement between the eastern and western regions, reflecting wider social and economic inequalities. Reducing the interregional education gap and improving the quality of education in the western region through policy interventions and resource allocation can contribute to social equity and overall progress, as well as provide balanced support for the development of human resources in the context of economic growth.

Table S1 Individual characteristics of respondents (China. 2023)

| Characteristic | Variable | Group | N | Mean | SD | Min | P50 | Max | Statistical Test | p-Value |
| --- | --- | --- | --- | --- | --- | --- | --- | --- | --- | --- |
| Gender | wordtest | Female | 3407 | 14.07 | 11.65 | 0 | 14 | 34 | t-test=-16.79 | 0.000*** |
|  |  | Male | 3293 | 18.54 | 10.07 | 0 | 21 | 34 |  |  |
|  | mathtest | Female | 3407 | 7.61 | 6.641 | 0 | 7 | 24 | t-test=-15.89 | 0.000*** |
|  |  | Male | 3293 | 10.09 | 6.066 | 0 | 10 | 24 |  |  |
| Age | wordtest | <= 50 | 3844 | 20.35 | 9.863 | 0 | 22 | 34 | t-test=38.45 | 0.000*** |
|  | mathtest | >50 | 2856 | 10.78 | 10.36 | 0 | 8 | 34 |  |  |
|  | wordtest | <= 50 | 3844 | 11.10 | 6.191 | 0 | 12 | 24 | t-test=36.44 | 0.000*** |
|  | mathtest | >50 | 2856 | 5.77 | 5.542 | 0 | 5 | 24 |  |  |
| Marriage | wordtest | Married | 5329 | 15.57 | 10.79 | 0 | 18 | 34 | t-test=-10.23 | 0.000*** |
|  |  | Unmarried | 1371 | 18.99 | 11.98 | 0 | 23 | 34 |  |  |
|  | mathtest | Married | 5329 | 8.24 | 6.047 | 0 | 8 | 24 | t-test=-15.02 | 0.000*** |
|  |  | Unmarried | 1371 | 11.13 | 7.525 | 0 | 12 | 24 |  |  |
| Education | wordtest | Illiterate and semi-illiterate | 2336 | 5.58 | 7.413 | 0 | 0 | 34 | Kruskal-Wallis test=3878.68 | 0.000*** |
|  |  | Elementary | 1378 | 16.59 | 8.443 | 0 | 18 | 34 |  |  |
|  |  | Middle | 1811 | 22.61 | 6.839 | 0 | 23 | 34 |  |  |
|  |  | High school | 769 | 26.55 | 5.338 | 0 | 28 | 34 |  |  |
|  |  | Undergraduate | 266 | 27.93 | 6.090 | 0 | 29 | 34 |  |  |
|  |  | Master | 133 | 30.56 | 3.199 | 19 | 31 | 34 |  |  |
|  |  | Doctor | 7 | 30.29 | 3.946 | 24 | 32 | 34 |  |  |
|  | mathtest | Illiterate and semi-illiterate | 2336 | 3.03 | 3.574 | 0 | 1 | 24 | Kruskal-Wallis test=4539.89 | 0.  000*** |
|  |  | Elementary | 1378 | 6.83 | 3.699 | 0 | 7 | 24 |  |  |
|  |  | Middle | 1811 | 12.50 | 3.699 | 0 | 12 | 24 |  |  |
|  |  | High school | 769 | 16.68 | 4.288 | 0 | 18 | 24 |  |  |
|  |  | Undergraduate | 266 | 17.22 | 4.152 | 0 | 18 | 24 |  |  |
|  |  | Master | 133 | 18.53 | 3.211 | 9 | 18 | 24 |  |  |
|  |  | Doctor | 7 | 20.71 | 2.563 | 18 | 20 | 24 |  |  |
| Health | wordtest | Very healthy | 851 | 18.48 | 10.39 | 0 | 21 | 34 | Kruskal-Wallis test=582.67 | 0.000*** |
|  |  | Well | 1221 | 18.81 | 10.62 | 0 | 21 | 34 |  |  |
|  |  | More healthy | 2444 | 17.94 | 10.86 | 0 | 21 | 34 |  |  |
|  |  | General | 865 | 15.53 | 10.60 | 0 | 18 | 34 |  |  |
|  |  | Unhealthy | 1319 | 9.86 | 10.38 | 0 | 7 | 34 |  |  |
|  | mathtest | Very healthy | 851 | 10.31 | 6.388 | 0 | 12 | 24 | Kruskal-Wallis test=604.34 | 0.000*** |
|  |  | Well | 1221 | 10.80 | 6.791 | 0 | 12 | 24 |  |  |
|  |  | More healthy | 2444 | 9.510 | 6.294 | 0 | 9 | 24 |  |  |
|  |  | General | 865 | 8.08 | 5.988 | 0 | 8 | 24 |  |  |
|  |  | Unhealthy | 1319 | 5.271 | 5.367 | 0 | 4 | 24 |  |  |
| Social status | wordtest | 1 | 617 | 16.15 | 11.26 | 0 | 19 | 34 | Kruskal-Wallis test=268.20 | 0.000*** |
|  |  | 2 | 1049 | 17.11 | 11.27 | 0 | 20 | 34 |  |  |
|  |  | 3 | 2944 | 17.50 | 11.07 | 0 | 21 | 34 |  |  |
|  |  | 4 | 1272 | 16.45 | 10.71 | 0 | 19 | 34 |  |  |
|  |  | 5 | 818 | 10.55 | 9.916 | 0 | 8 | 34 |  |  |
|  | mathtest | 1 | 617 | 8.47 | 6.500 | 0 | 8 | 24 | Kruskal-Wallis test=272.76 | 0.000*** |
|  |  | 2 | 1049 | 9.17 | 6.451 | 0 | 9 | 24 |  |  |
|  |  | 3 | 2944 | 9.63 | 6.541 | 0 | 9 | 24 |  |  |
|  |  | 4 | 1272 | 8.98 | 6.289 | 0 | 8 | 24 |  |  |
|  |  | 5 | 818 | 5.53 | 5.503 | 0 | 4 | 24 |  |  |
| Employment status | wordtest | Unemployed | 43 | 24.07 | 6.843 | 6 | 25 | 33 | Kruskal-Wallis test=717.40 | 0.000*** |
|  |  | Other status | 403 | 28.71 | 4.757 | 6 | 30 | 34 |  |  |
|  |  | Withdrawal from the labor market | 1365 | 13.00 | 11.21 | 0 | 12 | 34 |  |  |
|  |  | In employment | 4889 | 16.08 | 10.78 | 0 | 19 | 34 |  |  |
|  | mathtest | Unemployed | 43 | 12.28 | 4.404 | 1 | 12 | 24 | Kruskal-Wallis test=708.92 | 0.000*** |
|  |  | Other status | 403 | 17.42 | 4.845 | 0 | 18 | 24 |  |  |
|  |  | Withdrawal from the labor market | 1365 | 6.86 | 6.078 | 0 | 6 | 24 |  |  |
|  |  | In employment | 4889 | 8.64 | 6.155 | 0 | 8 | 24 |  |  |
| Region | wordtest | Eastern | 2380 | 17.02 | 10.47 | 0 | 20 | 34 | Kruskal-Wallis test=38.17 | 0.000*** |
|  |  | Central | 341 | 18.56 | 12.00 | 0 | 23 | 34 |  |  |
|  |  | Western | 3979 | 15.62 | 11.38 | 0 | 18 | 34 |  |  |
|  | mathtest | Eastern | 2380 | 9.20 | 5.999 | 0 | 9 | 24 | Kruskal-Wallis test=41.69 | 0.000*** |
|  |  | Central | 341 | 10.21 | 7.242 | 0 | 12 | 24 |  |  |
|  |  | Western | 3979 | 8.49 | 6.665 | 0 | 8 | 24 |  |  |
| Total | wordtest |  | 6700 | 16.27 | 11.13 | 0 | 19 | 34 |  |  |
|  | mathtest |  | 6700 | 8.828 | 6.484 | 0 | 8 | 24 |  |  |

Notes: *, **, and *** indicate significance at the 10%, 5%, and 1% levels, respectively. The same applies to the table below.


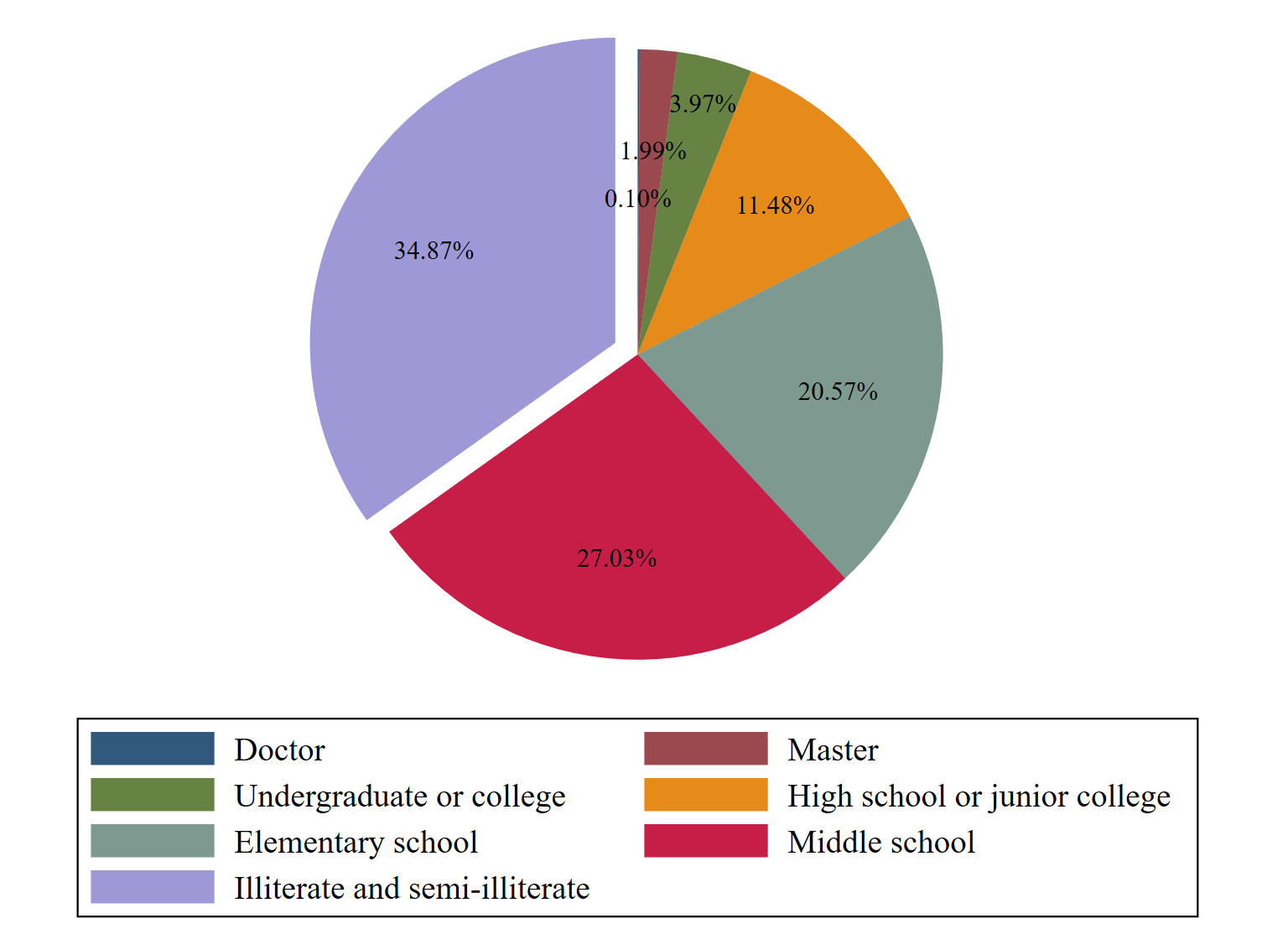


Figure S1 Highest education level of respondents (China. 2023)


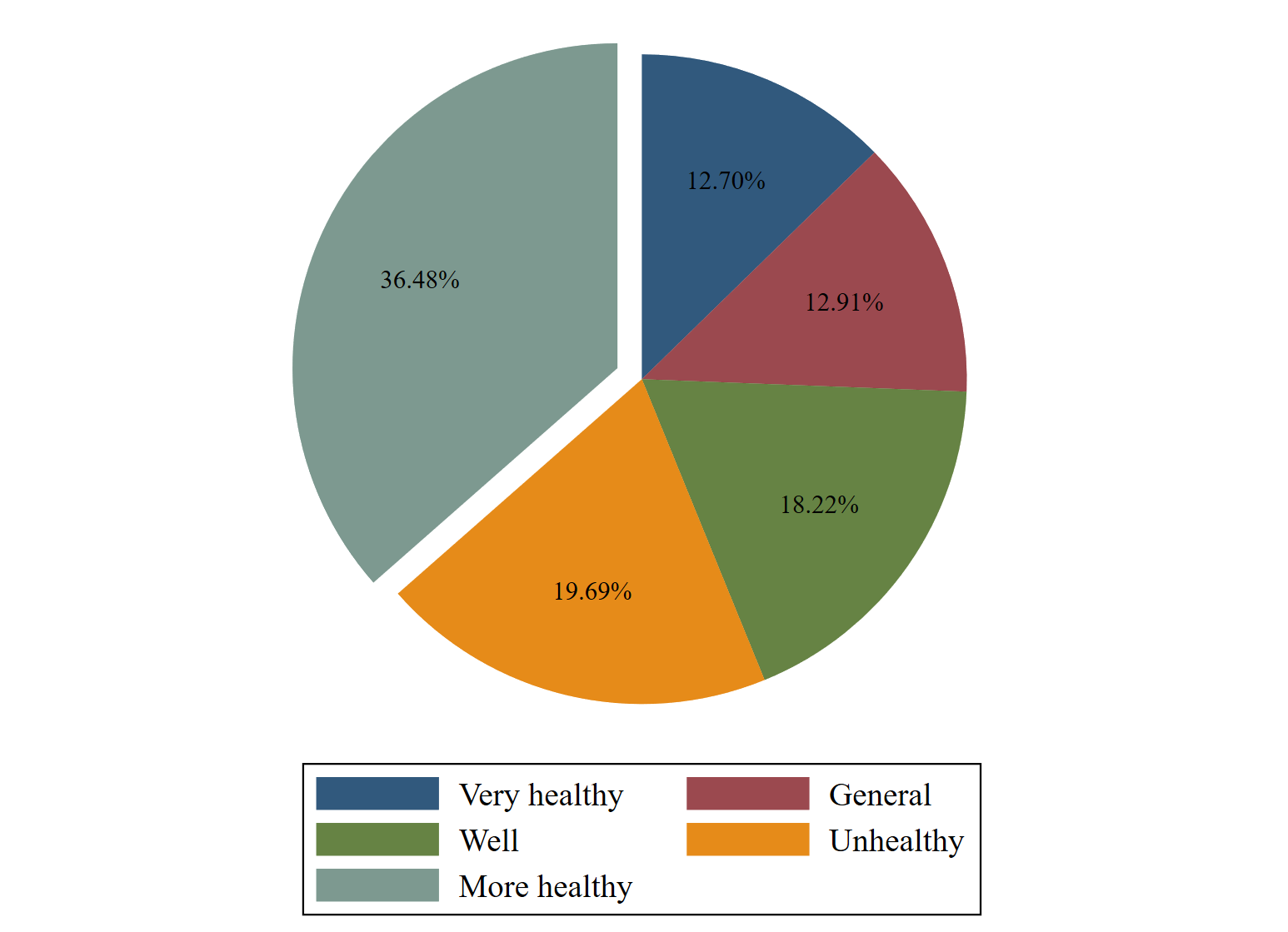


Figure S2 Health status of respondents (China. 2023)


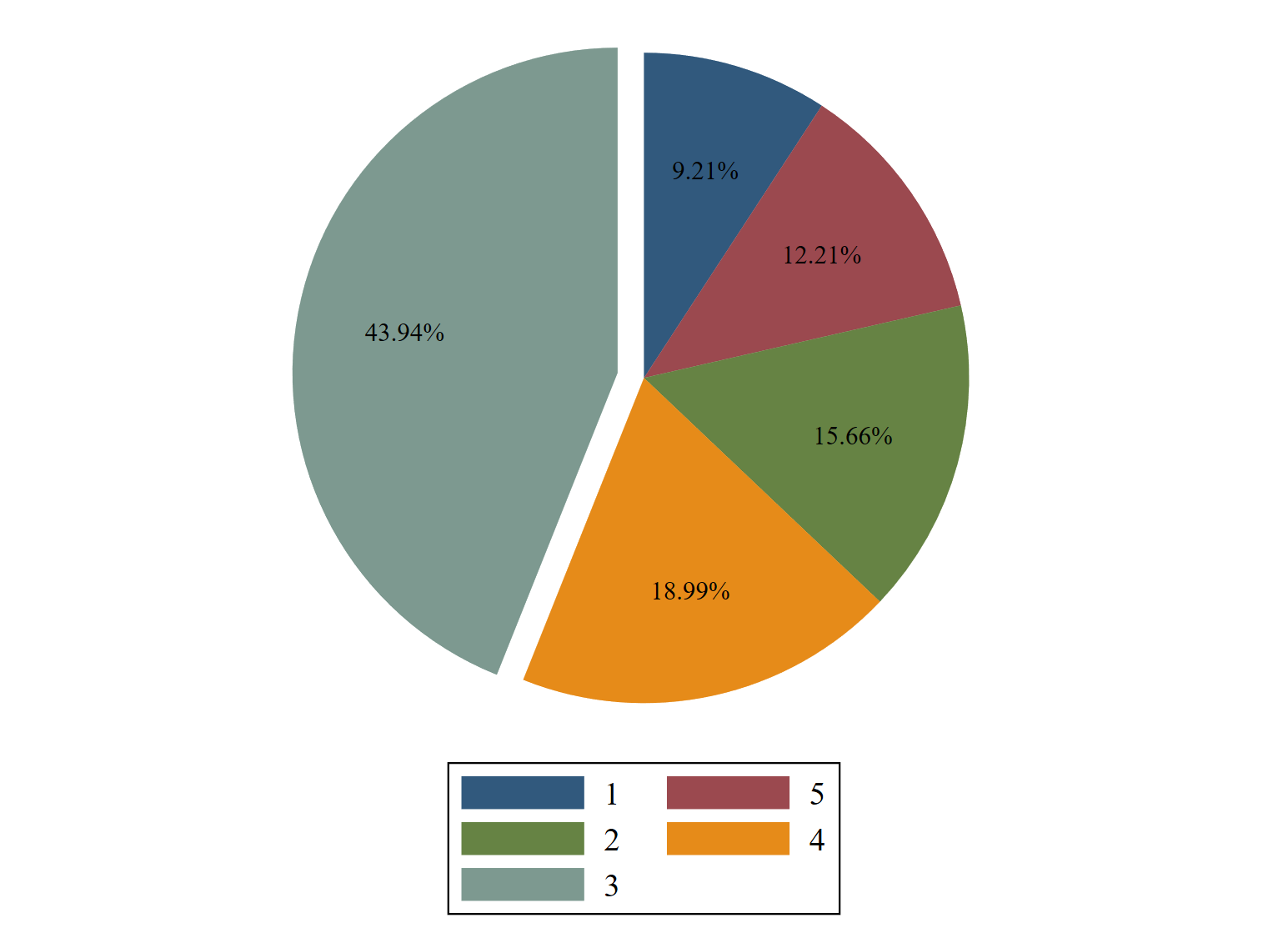


Figure S3 Social status of respondents (China. 2023)


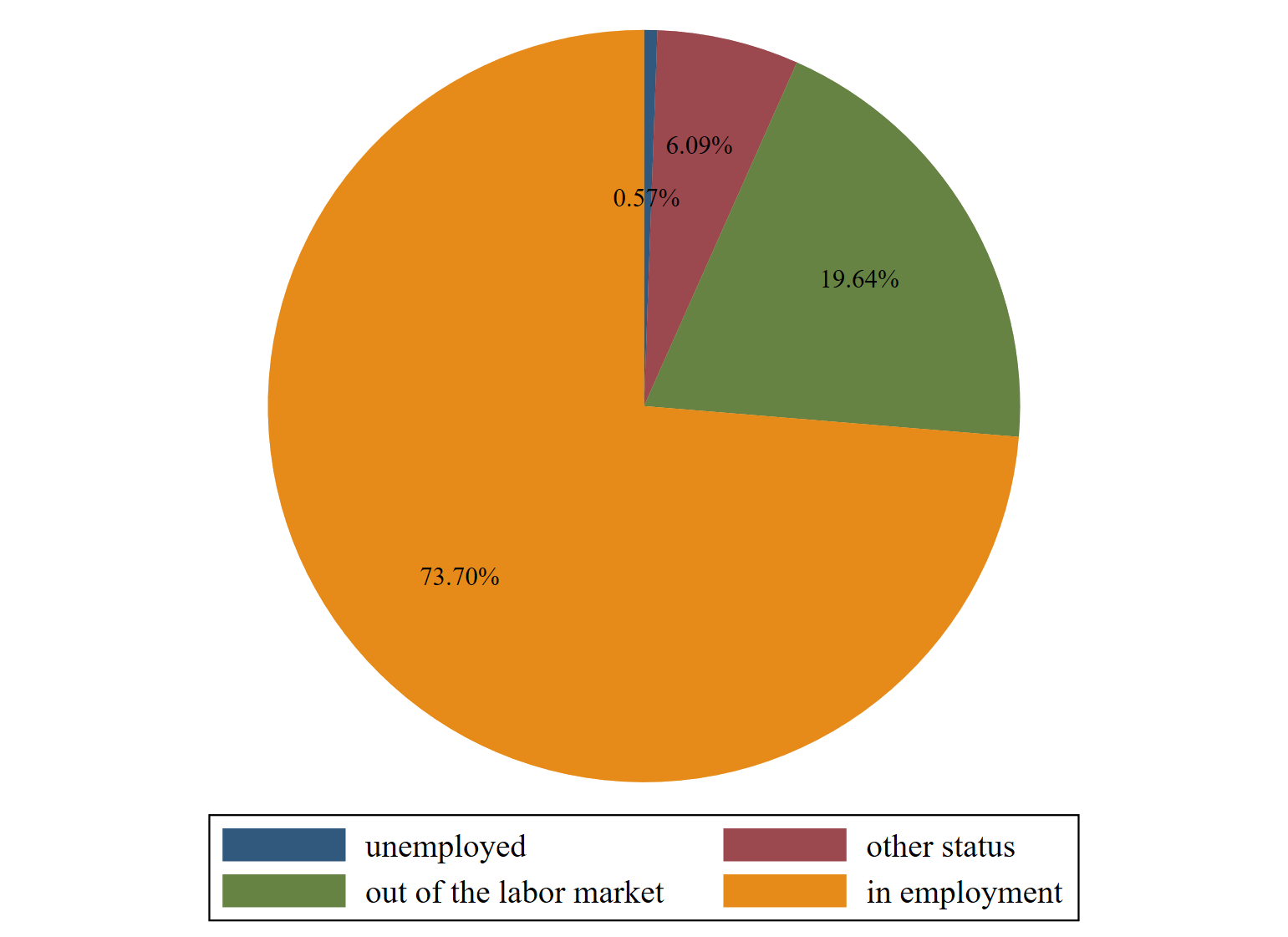


Figure S4 Employment status of respondents (China. 2023)


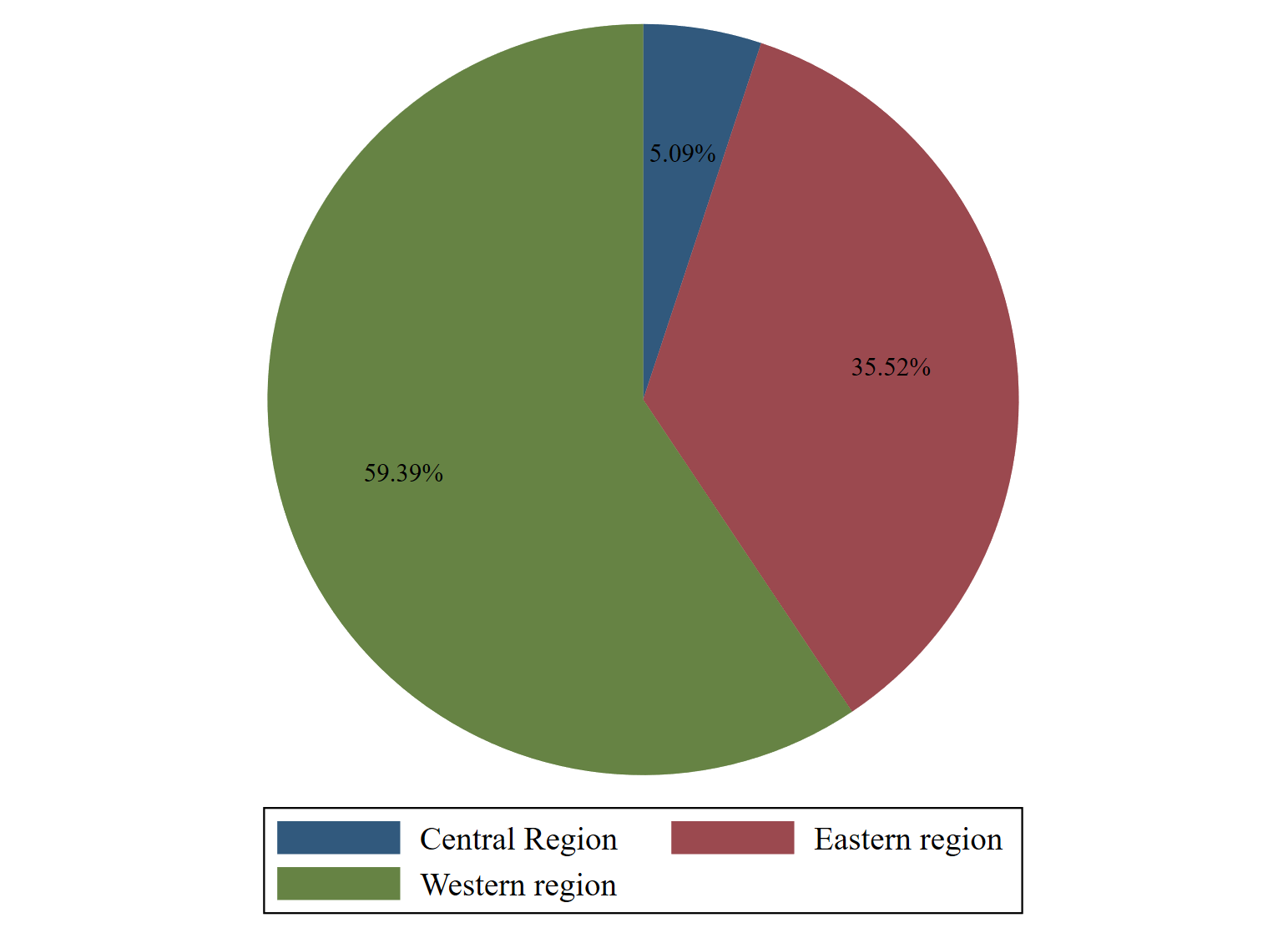


Figure S5 Percentage of respondents by region (China. 2023)

The main variables are defined in Supplementary File Table S2, and the descriptive statistics can be found in Table S3. The standardized Wordtest and Mathtest scores have means close to zero. Fire incident counts, categorized as Upfires and Nonupfires. Upfires has a mean of 3.133, a standard deviation of 6.260 and a maximum value of 32.5, while Nonupfires has a mean of 9, a standard deviation of 18.95 and a maximum value of 101.8. The number of wildfire shows great variability with a wide range of values.

Meteorological variables encompass precipitation, temperature, wind speed, and humidity. The precipitation displays notable variability, with a mean of 803.485 mm and a standard deviation of 495.700 mm, indicating a highly uneven distribution. Temperature exhibits a mean of 12.230°C and a standard deviation of 6.182°C, suggesting moderate variability. Wind speed and humidity present mean of 2.046 m/s and 65.456%, respectively, with relatively lower standard deviations (0.624 m/s for wind speed and 9.874% for humidity), indicating more consistent conditions. Demographic variables, including gender, income, status, and medical insurance, offer insights into the population's characteristics. Specifically, the income and status means are 10.260 and 3.093, respectively, and the standard deviations are 1.356 and 1.093, respectively, showing moderate means and standard deviations, suggesting that respondents are relatively homogeneous in terms of socio-economic status, with few extreme outliers. Air quality indicators comprise PM2.5, PM10, Air Quality Index (AQI), and NO2, all displaying relatively large average values, signifying a serious air pollution issue requiring further improvement.

Table S2 Variable Definitions (China. 2023)

| Panel A： Variable of interest | |
| --- | --- |
| Variable | Variable Descriptions |
| Wordtest | Word test scores were recorded based on the number of questions answered correctly by the respondents and were subsequently standardized. |
| Mathtest | Math test scores were recorded based on the number of correctly answered items by the participants and were subsequently standardized. |
| Upfires | The number of upwind wildfires within a 50-kilometer radius of the administrative center. Divide by 10. |
| Nonupfires | The number of non-upwind wildfires within a 50-kilometer radius of the administrative center. Divide by 10. |
| Panel B： Control variables | |
| Precipitation | The annual average precipitation; Unit: mm. |
| Temperature | The annual average temperature; Unit: °C. |
| Wind_speed | The annual average wind speed; Unit: m/s. |
| Humidity | The annual relative humidity; Unit: %. |
| Gender | Gender: Female=0, Male=1. |
| Income | Total family income, log-transformed; Unit: yuan. |
| Status | Surveyed individual’s social ranking in the local area: Very low=1, Very high=5. |
| Medical_Insurance | Medical health insurance: No=0, Yes=1. |
| Panel C： Mechanism Variables | |
| PM2.5 | The annual average PM2.5 concentration value takes the log; Unit: ug/m^3^. |
| AQI | The annual comprehensive air quality index takes the log. |
| PM10 | The annual average PM10 concentration value takes the log; Unit: ug/m^3^. |
| NO_2_ | The annual average NO_2_ concentration value takes the log; Unit: ug/m^3^. |

Table S3 Descriptive Statistics of Variables (China. 2023)

| Variable | Obs | Mean | SD | Min | Max |
| --- | --- | --- | --- | --- | --- |
| Wordtest | 6700 | -0.020 | 1.010 | -1.496 | 1.589 |
| Mathtest | 6700 | -0.003 | 1.032 | -1.408 | 2.411 |
| Upfires | 6700 | 3.133 | 6.260 | 0.000 | 32.500 |
| Nonupfires | 6700 | 9.000 | 18.950 | 0.200 | 101.800 |
| Precipitation | 6700 | 803.485 | 495.700 | 274.605 | 2062.300 |
| Temperature | 6700 | 12.230 | 6.182 | -1.819 | 22.654 |
| Wind_speed | 6700 | 2.046 | 0.624 | 1.037 | 3.825 |
| Humidity | 6700 | 65.456 | 9.874 | 47.138 | 82.154 |
| Gender | 6700 | 0.491 | 0.500 | 0.000 | 1.000 |
| Income | 6700 | 10.260 | 1.356 | 0.000 | 13.998 |
| Status | 6700 | 3.093 | 1.093 | 1.000 | 5.000 |
| Medical_Insurance | 6700 | 0.930 | 0.255 | 0.000 | 1.000 |
| PM2.5 | 6700 | 44.138 | 13.594 | 20.031 | 86.615 |
| PM10 | 6700 | 84.556 | 28.536 | 37.014 | 150.200 |
| AQI | 6700 | 72.128 | 19.056 | 37.289 | 124.201 |
| NO_2_ | 6700 | 31.067 | 11.320 | 13.894 | 58.451 |

**Robustness Checks**

The following robustness testing methodology shows that our baseline conclusions continue to hold.

**Alteration of the Dependent Variable**

In order to enhance the reliability of the research findings, this paper conducts robustness tests by selecting other dependent variables that measure cognitive ability. The specific results are shown in [Table S4](#TABLE4). Columns (1) and (2) of Table 4 use unstandardized test scores as proxies for cognitive ability. Columns (3) to (6) use the word and math test scores obtained according to the questionnaire algorithm of the year as the dependent variable. In particular, columns (3) and (4) alter the dependent variable to standardized test scores acquired based on the questionnaire algorithm of the year. Meanwhile, in columns (5) and (6), unstandardized test scores obtained according to the questionnaire algorithm of the year continue to be used as proxies for cognitive ability. All regression results indicate that after using different dependent variables to measure cognitive ability, it is still found that wildfire significantly reduces individual test scores.

**Substituting the Wildfire Counts with an Alternative Distance Range**

In the baseline regression, the explanatory variable is set as the number of wildfires within 50km of the administrative center. Following the design approach of Graff Zivin and Neidell [22], this paper further sets the explanatory variable as the number of wildfires within 40km or 60km of the administrative center. The empirical results are shown in Panel A of Table S5.

**Modifying the Angular Perspective for Wildfire Counts**

In our preceding analysis, our baseline model specification uses the angle of 45° to define upwind and downwind wildfires. In the present study, we alter the angle to 30 and 60°. The results of this regression analysis are presented in Panel B of Table S5.

**Analysis of the Impact of Fire Points with Varying Levels of Confidence**

This study builds upon the methodology employed by Rangel and Vogl [27] to examine the impact of wildfires with varying levels of confidence on cognitive performance. Confidence represents a metric used to measure the extent of thermal anomalies observed in fire points and serves as an indicator of the certainty associated with pixels identified as fire points. Algorithmic calculations classify fire points into high, normal, or low confidence levels. In this study, we conducted a regression analysis using only non-low confidence level wildfires (high and normal confidence levels). The results are presented in Panel A of Table S6.

**Analysis of the Effects of Wildfires on the Cognitive Performance of Non-Agricultural Respondents**

This paper examines whether there is an estimation bias in the effect caused by agricultural work respondents. Agricultural respondents may experience increased levels of fatigue or distraction during the harvest season, potentially impacting their performance on cognitive tests. Additionally, agricultural respondents may be more susceptible to exposure to wildfire, thereby exacerbating the negative effects of such hazards on their cognitive abilities. To control for these potential confounding factors, we restricted our analysis to respondents not engaged in agricultural activities. The results are shown in Panel B of Table S6.

**Adjustment of clustering standard errors**

The findings derived from our regression analysis, utilizing various clustering standard errors, are summarized in [Table S7](#TABLE7). Initially, we clustered the standard errors at both the household and county-year levels, with the results displayed in columns (1) and (2). Subsequently, we clustered the standard errors at both the household and city-year levels, with the results presented in columns (3) and (4). Our findings indicate that our baseline results remain unchanged.

**Adding control variables**

We consider that an individual’s age, education level, and health status may also affect an individual's cognitive ability, and therefore further add control variables such as Age, Education, and Health to the baseline model. The results are shown in Table S8, Panl A shows the results of the effect of wildfire on word test scores with the addition of control variables, and Panl B shows the results of the effect of wildfire on math test scores with the addition of control variables. The results all indicate that there is a significant negative effect of wildfire on Wordtest and Mathtest, consistent with the results of the baseline regression.

Table S4: Changing the Dependent Variable (China. 2023)

|  | (1) | (2) | (3) | (4) | (5) | (6) |
| --- | --- | --- | --- | --- | --- | --- |
|  | wordtest | mathtest | Wordtest2 | Mathtest2 | wordtest2 | mathtest2 |
| Upfires | -1.221*** | -0.623** | -0.118*** | -0.114** | -1.325*** | -0.654** |
|  | (-3.519) | (-2.139) | (-3.615) | (-2.210) | (-3.615) | (-2.210) |
| Nonupfires | 1.370*** | 0.858** | 0.129*** | 0.154** | 1.448*** | 0.882** |
|  | (3.420) | (2.520) | (3.463) | (2.522) | (3.463) | (2.522) |
| Upfires-Nonupfires | -2.591*** | -1.481** | -0.247*** | -0.268** | -2.773*** | -1.536** |
|  | (-3.582) | (-2.383) | (-3.648) | (-2.407) | (-3.648) | (-2.407) |
| Observations | 6700 | 6700 | 6700 | 6700 | 6700 | 6700 |
| R^2^ | 0.526 | 0.490 | 0.529 | 0.484 | 0.529 | 0.484 |
| Meteorological control variables | Y | Y | Y | Y | Y | Y |
| Individual control variables | Y | Y | Y | Y | Y | Y |
| Household FE | Y | Y | Y | Y | Y | Y |
| Province-Year FE | Y | Y | Y | Y | Y | Y |

Table S5: Empirical Analysis of the Impact of Wildfire Disaster on Cognitive Abilities (China. 2023)

|  | (1) | (2) | (3) | (4) |  |  |
| --- | --- | --- | --- | --- | --- | --- |
|  | Wordtest | Mathtest | Wordtest | Mathtest |  |  |
| Panel A | 40km | | 60km | |  |  |
| Upfires | -0.109*** | -0.090** | -0.096*** | -0.096** |  |  |
|  | (-3.522) | (-2.004) | (-3.478) | (-2.497) |  |  |
| Nonupfires | 0.134*** | 0.135** | 0.108*** | 0.133*** |  |  |
|  | (3.419) | (2.390) | (3.372) | (2.904) |  |  |
| Upfires-Nonupfires | -0.243*** | -0.225** | -0.204*** | -0.229*** |  |  |
|  | (-3.577) | (-2.256) | (-3.568) | (-2.784) |  |  |
| R^2^ | 0.526 | 0.490 | 0.526 | 0.490 |  |  |
| Panl B | 30° | | 60° | |  |  |
| Upfires | | | -0.268*** | -0.220* | -0.083*** | -0.079** |
|  | | | (-2.652) | (-1.763) | (-3.105) | (-1.995) |
| Nonupfires | | | 0.141** | 0.133** | 0.156*** | 0.185** |
|  | | | (2.529) | (1.976) | (3.121) | (2.338) |
| Upfires-Nonupfires | | | -0.409*** | -0.354* | -0.239*** | -0.264** |
|  | | | (-2.625) | (-1.846) | (-3.206) | (-2.254) |
| R^2^ | | | 0.526 | 0.490 | 0.526 | 0.490 |
| Observations | | 6700 | 6700 | 6700 | 6700 |  |
| Meteorological control variables | Y | Y | Y | Y |  |  |
| Individual control variables | N | Y | N | Y |  |  |
| Household FE | Y | Y | Y | Y |  |  |
| Province-Year FE | Y | Y | Y | Y |  |  |

Table S6: Empirical results of non-farm respondents' cognitive ability and non-low confidence wildfires (China. 2023)

|  | (1) | | (2) | (3) | (4) |
| --- | --- | --- | --- | --- | --- |
|  | Wordtest | | Mathtest | Wordtest | Mathtest |
| Panel A | | Effects of non-low confidence wildfires on cognitive performance | | | |
| Upfires | -0.086*** | | -0.099*** | -0.078* | -0.090** |
|  | (-2.884) | | (-3.384) | (-1.885) | (-2.187) |
| Nonupfires | 0.107*** | | 0.115*** | 0.124** | 0.132*** |
|  | (3.072) | | (3.366) | (2.562) | (2.709) |
| Upfires-Nonupfires | -0.193*** | | -0.214*** | -0.202** | -0.223** |
|  | (-3.123) | | (-3.520) | (-2.310) | (-2.527) |
| Observations | 6700 | | 6700 | 6700 | 6700 |
| R^2^ | 0.476 | | 0.526 | 0.444 | 0.490 |
| Panl B | | Effects of wildfires on the cognitive abilities of non-farm work respondents | | | |
| Upfires | -0.093*** | | -0.105*** | -0.088* | -0.099** |
|  | (-2.880) | | (-3.282) | (-1.878) | (-2.090) |
| Nonupfires | 0.114*** | | 0.121*** | 0.134** | 0.142** |
|  | (3.084) | | (3.304) | (2.480) | (2.554) |
| Upfires-Nonupfires | -0.207*** | | -0.226*** | -0.221** | -0.241*** |
|  | (-3.106) | | (-3.416) | (-2.246) | (-2.383) |
| R^2^ | 0.480 | | 0.531 | 0.450 | 0.496 |
| Observations | 6,465 | | 6,465 | 6,465 | 6,465 |
| Meteorological control variables | Y | | Y | Y | Y |
| Individual control variables | Y | | Y | Y | Y |
| Household FE | Y | | Y | Y | Y |
| Province-Year FE | Y | | Y | Y | Y |

Table S7: Regression Results Utilizing Varying Clustering Standard Errors (China. 2023)

|  | (1) | (2) | (3) | (4) |
| --- | --- | --- | --- | --- |
|  | The household and county-year levels | | The household and city-year levels | |
|  | Wordtest | Mathtest | Wordtest | Mathtest |
| Upfires | -0.111*** | -0.099** | -0.111*** | -0.099** |
|  | (-4.156) | (-2.505) | (-4.141) | (-2.497) |
| Nonupfires | 0.124*** | 0.137*** | 0.124*** | 0.137*** |
|  | (4.169) | (3.164) | (4.170) | (3.183) |
| Upfires-Nonupfires | -0.235*** | -0.236*** | -0.235*** | -0.236*** |
|  | (-4.261) | (-2.887) | (-4.254) | (-2.890) |
| Observations | 6700 | 6700 | 6700 | 6700 |
| R^2^ | 0.526 | 0.490 | 0.526 | 0.490 |
| Meteorological control variables | Y | Y | Y | Y |
| Individual control variables | N | N | N | N |
| Household FE | Y | Y | Y | Y |
| Province-Year FE | Y | Y | Y | Y |

Table S8: Adding control variables (China. 2023)

|  | (1) | (2) | (3) | (4) |
| --- | --- | --- | --- | --- |
| Panl A | Wordtest | Wordtest | Wordtest | Wordtest |
| Upfires | -0.058*** | -0.040*** | -0.095*** | -0.028*** |
|  | (-4.646) | (-5.057) | (-14.035) | (-3.422) |
| Nonupfires | 0.067*** | 0.044*** | 0.114*** | 0.033*** |
|  | (4.673) | (4.726) | (14.384) | (3.214) |
| Upfires-Nonupfires | -0.125*** | -0.083*** | -0.209*** | -0.061*** |
|  | (-4.688) | (-4.932) | (-14.37) | (-3.336) |
| Age | -0.034*** |  |  | -0.019*** |
|  | (-18.271) |  |  | (-15.921) |
| Education |  | 0.555*** |  | 0.404*** |
|  |  | (26.879) |  | (21.779) |
| Health |  |  | -0.181*** | -0.011 |
|  |  |  | (-9.766) | (-1.044) |
| Observations | 6700 | 6700 | 6700 | 6700 |
| R^2^ | 0.690 | 0.730 | 0.555 | 0.767 |
| Panl B | Mathtest | Mathtest | Mathtest | Mathtest |
| Upfires | -0.042 | -0.013*** | -0.081*** | -0.003 |
|  | (-1.440) | (-6.087) | (-4.271) | (-0.944) |
| Nonupfires | 0.075** | 0.038*** | 0.125*** | 0.029*** |
|  | (2.288) | (11.054) | (5.714) | (8.856) |
| Upfires-Nonupfires | -0.117* | -0.051*** | -0.207*** | -0.032*** |
|  | (-1.895) | (-9.867) | (-5.064) | (-5.267) |
| Age | -0.036*** |  |  | -0.016*** |
|  | (-16.675) |  |  | (-14.215) |
| Education |  | 0.675*** |  | 0.544*** |
|  |  | (41.124) |  | (44.197) |
| Health |  |  | -0.201*** | -0.017* |
|  |  |  | (-9.145) | (-1.950) |
| Observations | 6700 | 6700 | 6700 | 6700 |
| R^2^ | 0.670 | 0.778 | 0.525 | 0.805 |
| Meteorological control variables | Y | Y | Y | Y |
| Other individual control variables | Y | Y | Y | Y |
| Household FE | Y | Y | Y | Y |
| Province-Year FE | Y | Y | Y | Y |
